# Endothelial cell protein C receptor-mediated redistribution and tissue-level accumulation of factor VIIa

**DOI:** 10.1111/j.1538-7836.2012.04917.x

**Published:** 2012-11

**Authors:** C A Clark, R Vatsyayan, U Hedner, C T Esmon, U R Pendurthi, L V M Rao

**Affiliations:** * Department of Cellular and Molecular Biology, Center for Biomedical Research, The University of Texas Health Science Center at TylerTyler, TX, USA; † Department of Medicine, Malmö University Hospital, University of LundMalmö, Sweden; ‡ Cardiovascular Biology Research Program, Oklahoma Medical Research Foundation, Howard Hughes Medical InstituteOklahoma City, OK, USA

**Keywords:** endothelial cell protein C receptor, factor VIIa, hemophilia, hemostasis, tissue factor, transport

## Abstract

**Background:**

Recent studies show that activated factor VII (FVIIa) binds to the endothelial cell protein C receptor (EPCR) on the vascular endothelium; however, the importance of this interaction in hemostasis or pathophysiology is unknown.

**Objective:**

The aim of the present study was to investigate the role of the FVIIa interaction with EPCR on the endothelium in mediating FVIIa transport from the circulation to extravascular tissues.

**Methods:**

Wild-type, EPCR-deficient or ECPR-over-expressing mice were injected with human recombinant (r)FVIIa (120 μg kg^−1^ body weight) via the tail vein. At varying time intervals after rFVIIa administration, blood and various tissues were collected to measure FVIIa antigen and activity levels. Tissue sections were analyzed by immunohistochemistry for FVIIa and EPCR.

**Results:**

The data reveal that, after intravenous (i.v.) injection, rFVIIa rapidly disappears from the blood and associates with the endothelium in an EPCR-dependent manner. Immunohistochemical analyses revealed that the association of FVIIa with the endothelium was maximal at 30 min and thereafter progressively declined. The FVIIa association with the endothelium was undetectable at time points exceeding 24 h post-FVIIa administration. The levels of rFVIIa accumulated in tissue correlate with expression levels of EPCR in mice and FVIIa associated with tissues remained functionally active for periods of at least 7 days.

**Conclusions:**

The observation that an EPCR-dependent association of FVIIa with the endothelium is most pronounced soon after rFVIIa administration and subsequently declines temporally, combined with the retention of functionally active FVIIa in tissue homogenates for extended periods, indicates that FVIIa binding to EPCR on the endothelium facilitates the transport of FVIIa from circulation to extravascular tissues where TF resides.

## Introduction

Initiation of the coagulation cascade requires the binding of circulating activated factor VII (FVIIa) from plasma with tissue factor (TF) that is primarily localized on cells in extravascular tissue. There is ample evidence for the occurrence of continuous basal activation of TF–FVIIa-dependent coagulation in healthy individuals [1–3], indicating that traces of FVII comes in contact with TF even in the absence of vessel wall injury. Although a number of previous studies have suggested that physiologically active TF circulates in blood at meaningful concentrations, a careful analysis has revealed no detectable TF activity or antigen in blood from healthy individuals [4]. Therefore, it is unclear at present how FVII or FVIIa from the circulation comes in contact with TF in the absence of any vessel wall injury.

Recombinant (r)FVIIa has been demonstrated to be an effective hemostatic agent for the treatment of severe hemophilia patients with inhibitors against FVIII or FIX [5]. Recent studies suggest that prophylactic treatment of hemophiliacs or FVII-deficient patients with rFVIIa is effective in preventing bleeding disorders in these patients [6–12]. Interestingly, it was found that daily injection of rFVIIa for 3 months not only decreased significantly the number of mild-moderate joint bleeds during the treatment period but also reduced the number of hemorrhagic episodes during the 3-month post-treatment period [12]. Given the short biological half-life of rFVIIa in the circulation, it is unclear why regular administration once daily of rFVIIa reduces the number of hemorrhagic events in the post-treatment period. However, the data herein raise the possibility that rFVIIa administered to patients could be retained for a longer period perhaps in the extravascular space than indicated by the plasma half-life. At present, it is unknown whether a specific mechanism exists for the transport of FVIIa from the circulation to the extravasculature space.

Recent studies from us [13] and others [14,15] have established that FVIIa also binds in a true ligand-receptor fashion to the endothelial protein C receptor (EPCR), a major constituent of the protein C/activated protein C (PC/APC) anticoagulant pathway. The interaction between FVIIa and EPCR is capable of not only eliciting protease activated receptor-1 (PAR1)-mediated barrier protective signaling [16], but also promotes internalization of this receptor–ligand complex [17]. Our recent studies showed that rFVIIa administered intravenously (i.v.) to mice associates with the vascular endothelium after its administration and thereafter enters into perivascular tissues [17,18]. Although we have recently shown that human rFVIIa administered to mice specifically binds to EPCR on the endothelium [19], the importance of this interaction in facilitating the transport of rFVIIa from circulation into tissues is unknown.

The aim of present study was to investigate the contribution of EPCR on the vascular endothelium in the temporal sequestration, transport and extravascular compartmentalization of rFVIIa administered to mice. The data provided within this report show that soon after i.v. injection rFVIIa rapidly associates with EPCR on the vascular endothelium and EPCR promotes the accumulation of rFVIIa in tissues. Interestingly, sustained, functionally active rFVIIa persists in tissues for periods of time far exceeding its circulatory half-life and its association with the endothelium. Overall, our data indicates that EPCR is capable of modulating circulating FVIIa levels via sequestration to the vascular endothelium, transcytosis and accumulation in extravascular tissue. These findings establish a novel mechanism by which circulating FVIIa can be redistributed to tissues, allowing for prolonged retention and interaction with extravascular mediators including TF. This mechanism by which rFVIIa can be retained in tissues for extended periods of time far exceeding its circulatory half-life assumes substantial clinical relevance as rFVIIa is used for the management of hemophilia and other hemorrhagic bleeding conditions.

## Methods

### Reagents

Human rFVIIa was obtained from Novo Nordisk A/S (Maaloev, Denmark). Affinity purified polyclonal antibodies against human FVIIa were provided by Walter Kisiel (University of New Mexico, Albuquerque, NM, USA). The preparation of goat polyclonal antibodies to the recombinant soluble murine EPCR was described earlier [20]. Antigen retrieval solution, rabbit anti-goat secondary antibody and substrates used for immunohistochemistry were obtained from Dako North America (Carpinteria, CA, USA). Goat anti-rabbit secondary antibody was obtained from Sigma (St. Louis. MO, USA).

### Mice

The generation of EPCR-deficient mice (*Procr*^*−/−*^) and EPCR-over-expressing mice (Tie2-EPCR) were described earlier [21,22]. Either littermates of EPCR-deficient mice (when the experimental group contained both EPCR-deficient mice and EPCR-over-expressing mice) or C57Bl/6 mice (when the experimental group is limited to EPCR over expressing mice) obtained from Jackson Laboratory (Bar Harbor, ME, USA) were used as wild-type mice. All studies involving animals were conducted in accordance with the animal welfare guidelines set forth in the Guide for the Care and Use of Laboratory Animals and Department of Health and Human Services. All animal procedures were approved by the Institutional Animal Use and Care Committee.

### Administration of FVIIa to mice, collection of blood and tissues

Human rFVIIa was administered into anesthetized EPCR-deficient, wild-type littermate or EPCR-over-expressing mice via the tail vein at a dose of 120 μg kg^−1^ body weight in 100 μL of Tris-buffered saline (TBS, 50 mm Tris–HCl, 0.15 m NaCl, pH 7.5). At relevant time intervals, for example 30 min, 3, 8, 24 h, 3 or 7 days after rFVIIa administration, blood was collected via cardiac puncture in 1/10 volume 0.13 m sodium citrate anticoagulant buffer to measure human FVIIa levels in circulation. Mice were subsequently exsanguinated by severing the renal artery and perfused by flushing ice-cold saline containing 5 mm CaCl_2_ + 1 mm MgCl_2_ through the heart. In order to quantify tissue-level bioavailability of FVIIa after its administration, various organs (skin, lung, liver, kidney and bone joints) were collected after the vascular perfusion and exsanguination, tissues were briefly washed in ice-cold saline containing 5 mm CaCl_2_ + 1 mm MgCl_2_ and either fixed in Excel fixative (American Master*tech Scientific Inc., Lodi, CA, USA) for immunohistochemical processing or frozen at −80 °C for antigen/activity analyses. Bone joints used for immunohistochemistry were decalcified after the fixation process in EDF decal solution (Statlab Medical Products, McKinney, TX, USA).

### FVII antigen and activity assays

Plasma was collected as described above. Tissue homogenates were prepared by adding 0.5 mL of homogenization buffer (0.01 m Tris, 0.15 m NaCl, pH 7.4) per 100 mg of tissue and homogenized using a tissue homogenizer (Ultra Turrax, Model #SDT 1810; Tekmar company, Cincinnati, OH, USA) for 10–20 s. Tissue homogenates were incubated with 20 mm EDTA for 2 min at room temperature and then spun at 2650 *g* for 10 min in order to collect a TF-free supernatant containing putative rFVIIa. For analysis of FVIIa antigen and activity levels in bone joints, the above procedure was slightly modified to avoid having an unworkable, viscous homogenate. Bone joint sections were first finely cut and directly collected into EDTA-containing solution, freeze-thawed, vortexed, centrifuged and the supernatant was collected.

The amount of tissue-associated human FVIIa was determined in a human FVII-specific ELISA using rabbit anti-human FVIIa as the capture antibody and biotinylated rabbit anti-human FVIIa as the detecting antibody. The lower detection limit of the assay was approximately 1 ng mL^−1^ human FVIIa. FVIIa clotting activity was measured in a FVIIa-specific clotting assay as described previously [23] with minor modifications. Briefly, prothrombin time (PT) of diluted plasma and tissue samples was measured in a STart clotting machine (Diagnostica Stago, Parsippany, NJ, USA) using 1 mm PC/PS/PE vesicles containing 100 nm soluble TF, FVII-depleted plasma (George King, Bio-Medical, Overland Park, KS, USA) and 25 mm CaCl_2_ to initiate clotting after a 3-min warm-up period at 37 °C. In the absence of soluble TF, tissue samples gave similar prolonged clot times as of buffer blank, indicating that tissue supernatants are devoid of TF capable of interfering in the assay. For both the antigen and activity assays, human rFVIIa standards were prepared in tissue supernatants from un-injected mice. Plasma levels of FVIIa are reported as ng mL^−1^ of plasma; all antigen and activity levels in tissues were normalized to ng mg^−1^ of tissue protein.

### Immunohistochemistry

After overnight fixation, tissues were processed using graded alcohol followed by xylene and then embedded in paraffin using standard tissue processing procedures. Thin sections (5 μm) were prepared and processed for immunohistochemistry as described recently by our laboratory [18].

## Results

### Correlation between EPCR expression and circulatory levels of rFVIIa

**Fig. 1:**
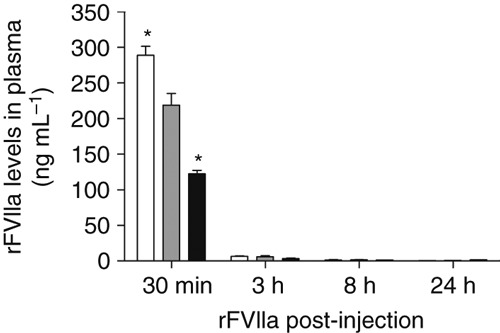
Endothelial protein C receptor (EPCR) lowers recombinant activated factor VII (rFVIIa) levels in the circulation after the administration of rFVIIa. EPCR-deficient, wild-type littermates or EPCR-over-expressing mice were injected intravenously via the tail vein with unlabeled FVIIa (120 μg kg^−1^ body weight). FVIIa levels in plasma derived from mice at varying time periods after rFVIIa administration were determined using a human FVII-specific ELISA. The data were shown as ng FVIIa present in plasma. Open bar, EPCR-deficient mice; grey bar, wild-type mice; black bar, EPCR-over-expressing mice (*n* = 6–7 for 30 min and 3 h time points, 2 for 8 and 24 h time points). *Indicates the value differs in a statistically significant manner when compared with the corresponding value noted for wild-type mice (*P* ≤ 0.05).

In our previous studies [13,17], we have shown that FVIIa is capable of binding to EPCR on endothelial cells *in vitro* and that this interaction results in internalization of the ligand–receptor complex. In the present study, we have sought to define the role of this interaction in the bioavailability of FVIIa *in vivo*. Therefore, we have investigated the effect of EPCR expression on the distribution of FVIIa in circulation as well as tissues at various time points after i.v. injection. Since we recently found that human FVIIa and not mouse FVIIa binds to EPCR effectively [19], we used human rFVIIa in the present study. In line with earlier pharmacokinetic studies [24,25], rFVIIa recovered in plasma was substantially lower than predicted soon after its administration in all genotypes. FVIIa activity levels in plasma at 30 min after the injection were about 10% of the injected concentration. More importantly, plasma FVIIa levels at 30 min was inversely correlated with EPCR expression (Fig. 1). At 30 min, rFVIIa levels in EPCR-over-expressing mice were approximately 55% of that which was found in wild-type mice, whereas levels in EPCR-deficient mice were approximately 32% higher compared with wild-type (Fig. 1). At 3 h, rFVIIa levels in circulation were < 1% of rFVIIa that was administered and no significant differences were observed in rFVIIa levels in plasma between the wild-type and EPCR transgenic mice at 3 h or later time points. This later observation was consistent with our recent data that showed bulk clearance of rFVIIa from blood was independent of EPCR [25].

### EPCR-dependent vascular sequestration of rFVIIa

**Fig. 2:**
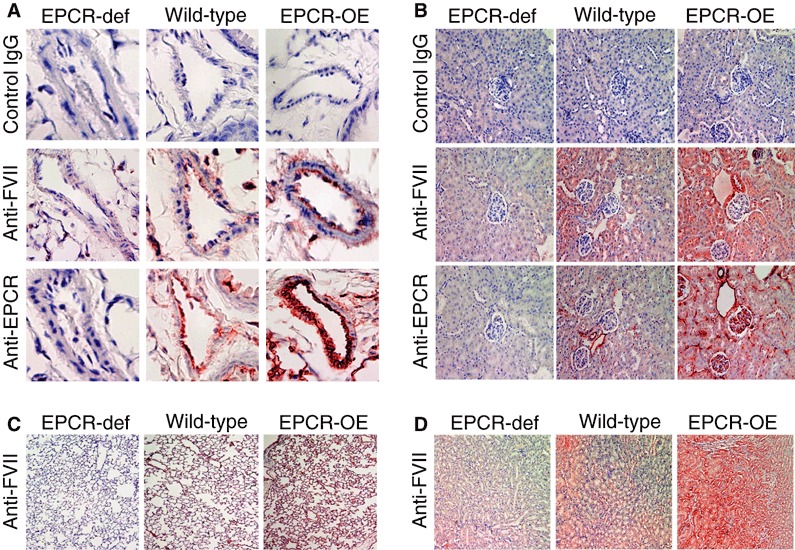
Endothelial protein C receptor (EPCR)-dependent binding of activated factor VII (FVIIa) to the endothelium. Human recombinant (r)FVIIa (120 μg kg^−1^) was injected to wild-type, EPCR-deficient (EPCR-def) or EPCR-over-expressing (EPCR-OE) mice via the tail vein. Mice were killed 30 min after the injection and perfused with 15 mL of ice-cold saline (supplemented with 5 mM CaCl_2_ and 1 mm MgCl_2_). Various tissues were collected and fixed in Excel Plus fixative and processed for tissue sectioning and immunohistochemistry. Staining of tissue sections for human FVIIa and EPCR were shown here. (A) The lungs (400× magnification), (B) kidney (400× magnification), (C) lungs (100× magnification) and (D) kidney (100× magnification).

Next, to determine if sequestration of rFVIIa by EPCR on the vascular endothelium could be a reason for the observed differences in rFVIIa levels in the circulation among various genotypes at 30 min after rFVIIa administration, we evaluated the extent of the rFVIIa association with the endothelium at 30 min in wild-type, EPCR-deficient and in EPCR-over-expressing mice. As shown in Fig. 2A, the staining for rFVIIa on the vascular endothelium of the lung is very intense in EPCR-over-expressing mice compared with wild-type. The rFVIIa association with the endothelium in EPCR-deficient mice was negligible. Analysis of kidney sections revealed that EPCR-dependent rFVIIa staining is associated with and around vascular cells of the cortical region (Fig. 2B). We have also observed an EPCR-dependent association of rFVIIa in other tissues too (e.g. bone joints, liver and skin), particularly on the endothelial lining of blood vessels. However, the intensity of rFVIIa staining among EPCR-deficient, wild-type and EPCR-over-expressing mice varied from tissue to tissue and was not as persistently robust as observed with the lung (data not shown). Although an EPCR-dependent association of FVIIa is clearly visible with kidney and liver, a FVIIa association with vessel wall endothelium is not as conspicuous as noted in the vascular bed of the lung. Furthermore, in the case of the kidney, structural heterogeneity may contribute to a variation in FVIIa staining in certain regions compared with global antigen and activity levels.

### EPCR-dependent accumulation of rFVIIa in tissues

**Fig. 3:**
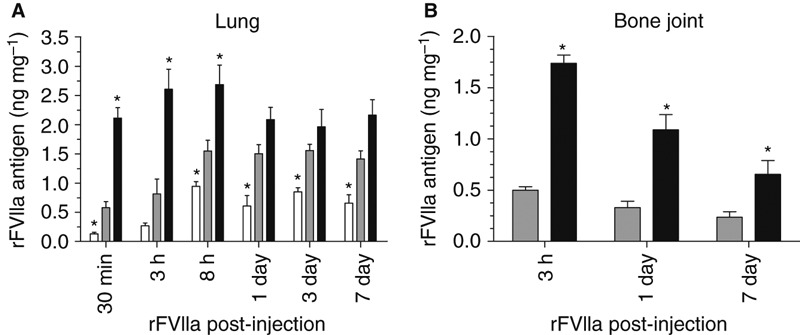
Endothelial protein C receptor (EPCR)-dependent accumulation of recombinant activated factor VII (rFVIIa) in the lung and bone joints. EPCR-deficient, wild-type littermates or EPCR-over-expressing mice were injected with human rFVIIa (120 μg mL^−1^) intravenously via the tail vein. Mice were killed at varying time intervals after rFVIIa administration and perfused with 15 mL of ice-cold saline supplemented with 5 mm CaCl_2_ and 1 mm MgCl_2_. FVIIa antigen levels in lung tissue extracts (A) and bone joints (B) were determined using human FVII-specific ELISA. Open bar, EPCR-deficient mice; gray bar, wild-type mice; black bar, EPCR-over-expressing mice (*n* = 3–8). *Indicates the value differs in a statistically significant manner when compared with the corresponding value noted for wild-type mice (*P* ≤ 0.05).

To determine whether EPCR expression on the endothelium regulates the entry of rFVIIa into tissues, we measured rFVIIa antigen in tissue extracts harvested at various intervals after rFVIIa administration in wild-type, EPCR-deficient and EPCR-over-expressing mice. At 30 min, rFVIIa levels in lung tissue homogenates from EPCR-deficient mice were only 23% of that which were found in wild-type mice, whereas levels were nearly four-fold higher in EPCR-over-expressing mice (Fig. 3A). Not only is an EPCR-dependent accumulation of rFVIIa observed at 30 min, but at all times points measured in this study. Even at 7 days after administration, rFVIIa was significantly retained in tissues and these accumulated FVIIa levels varied in an EPCR-dependent manner. rFVIIa levels in lung tissue homogenates of EPCR-deficient mice were significantly lower at all time points, including at 7 days where levels were approximately half of those seen in wild-type (*P* < 0.01). Tissue-level rFVIIa was considerably elevated in tissue homogenates of EPCR-over-expressing mice at all time points relative to wild-type and EPCR-deficient mice, in spite of a plateau effect at later times points (i.e. 1–7 days), and all mice displayed a significant retention of rFVIIa levels even 7 days after rFVIIa administration. In bone joints, similar to that observed in lungs, EPCR over-expression resulted in more significant accumulation of rFVIIa and this effect perpetuated for periods of time up to 7 days (Fig. 3B). At 3 h after rFVIIa administration, rFVIIa levels in bone joint isolates were approximately 3.5-fold higher than wild-type mice (*P* < 0.01). Similar to rFVIIa levels in lung homogenates, levels slightly regressed at extended time points, but even at 1 and 7 days, levels in bone joint isolates of EPCR-over-expressing mice were approximately 3.3- and 2.8-fold higher, respectively, than the wild-type (*P* = 0.03 and *P* = 0.09, respectively). Altogether these results suggest a role for EPCR in the prolonged association of FVIIa with tissues. Although we observed a clear EPCR-dependent staining of FVIIa in the kidney (Fig. 2B), we could not detect significant differences in FVIIa antigen levels among EPCR-deficient, wild-type and EPCR-over-expressing mice. Structural heterogeneity may contribute to a variation in FVIIa staining in certain regions compared with antigen measurement with the entire tissue.

### Time dependence of the FVIIa association with the vascular endothelium of EPCR-transgenic mice

A positive correlation between EPCR expression and rFVIIa levels measured in tissue extracts even after many days after rFVIIa administration indicate that rFVIIa bound to EPCR on the endothelium may exit the vasculature and enter tissues. However, one possibility is that sequestered rFVIIa on the vascular endothelium may in fact remain for prolonged periods of time and it is this portion of rFVIIa that is contributing to what is being measured as tissue-associated rFVIIa. To determine whether or not FVIIa is effectively leaving the vasculature and entering tissues as opposed to remaining on the endothelium, we performed a series of time-course immunohistochemical analyses where an endothelial association of rFVIIa was determined at various stages after i.v. injection of rFVIIa.

**Fig. 4:**
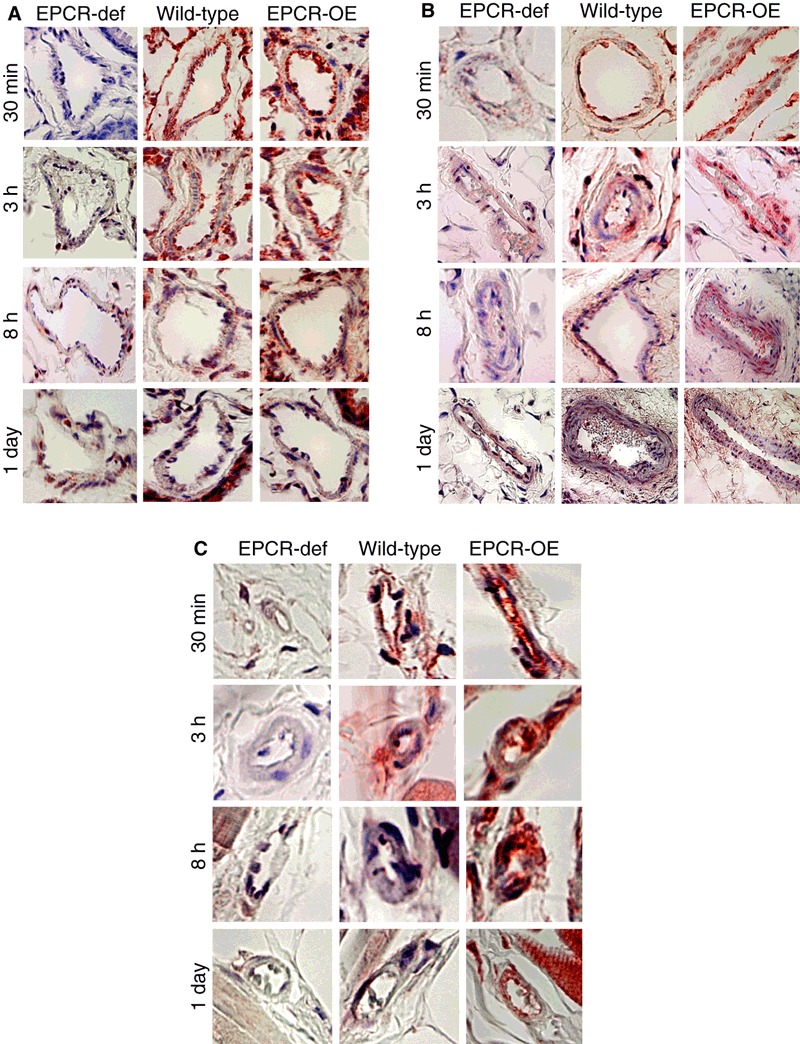
Recombinant activated factor VII (rFVIIa) associated with endothelium decreases in a time-dependent manner. Human rFVIIa (120 μg kg^−1^) was injected to wild-type, endothelial protein C receptor (EPCR)-deficient (EPCR-def) or EPCR-over-expressing (EPCR-OE) mice via the tail vein. Mice were killed at varying time intervals, 30 min to 1 day after rFVIIa administration, and perfused with ice-cold saline supplemented with 5 mm CaCl_2_ and 1 mm MgCl_2_. Various tissues were collected and fixed in Excel Plus fixative and processed for tissue sectioning and immunohistochemistry. (A) Lung, (B) bone joints and (C) skin (400× magnification).

As shown in Fig. 4 and as seen earlier (Fig. 2A), there is a considerable endothelial association of rFVIIa in wild-type mice, and to a more significant extent in EPCR-over-expressing mice, at 30 min after rFVIIa administration. A rFVIIa association with the endothelium of EPCR-deficient mice is minor compared with wild-type and EPCR-over-expressing mice as seen in the vessels of bone joints and skin (Fig. 4B,C). The very visible staining of rFVIIa on the vascular endothelium observed at 30 min in the wild-type mice progressively reduces at later time-points to the extent that at 1 day rFVIIa staining on the endothelium is in effect absent. The intense staining rFVIIa observed on the endothelium of EPCR-over-expressing mice at 30 min was markedly reduced at later time points. Also evident is that a peri- and extravascular association of rFVIIa with the lung, bone joint and skin vessels remains more perceptible for somewhat longer periods of time in EPCR-over-expressing mice (Fig. 4A–C). The time-wise comparison of immunohistochemical analysis of rFVIIa and the measurement of rFVIIa antigen levels in tissue extracts indicate that rFVIIa bound to the endothelium is effectively transcytosed and redistributed to tissues where it is retained for prolonged periods of time. Diffuse distribution of low, albeit potentially physiologically significant, levels of rFVIIa in extravascular tissues make it difficult to visualize rFVIIa staining in this compartment.

### rFVIIa accumulated in tissue is functionally active

**Fig. 5:**
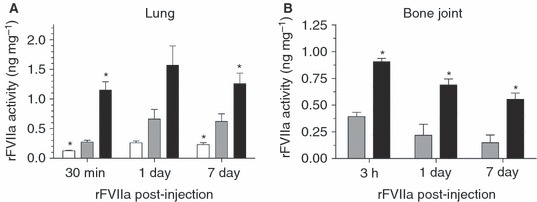
Recombinant activated factor VII (rFVIIa) transcytosed into extravascular tissue via the endothelial protein C receptor (EPCR)-dependent mechanism is functionally active. EPCR-deficient (open bar), wild-type littermates (gray bar) or EPCR-over-expressing mice (black bar) were injected with human rFVIIa (120 μg kg^−1^ body weight) intravenously via the tail vein. Mice were killed at varying time intervals after rFVIIa administration and perfused with 15 mL of ice-cold saline supplemented with 5 mm CaCl_2_ and 1 mm MgCl_2_. FVIIa activity levels in lung tissue extracts (A) and bone joints (B) were determined using a FVIIa-specific coagulant assay. (A) Lung tissue extracts and (b) bone joint isolates (*n* = 3–6). *Indicates the value differs in a statistically significant manner when compared with the corresponding value noted for wild-type mice (*P* ≤ 0.05).

To assess the potential significance of FVIIa antigen accumulated in tissues in hemostasis, we determined whether FVIIa redistributed to tissues is functionally active. The measurement of FVIIa-specific coagulant activity of tissue extracts revealed that a majority of rFVIIa redistributed to tissues retained its coagulant activity (Fig. 5). The coagulant active rFVIIa levels measured in lung and bone joint tissue isolates obtained at varying time intervals varied in a statistically significant and EPCR-dependent manner. At 30 min, 1, and 7 days after rFVIIa administration, rFVIIa levels in lung homogenates from EPCR-deficient mice were 45.3%, 39.5% and 37.0% of those in wild-type control mice. EPCR-over-expressing mice had dramatically elevated levels of coagulant-active rFVIIa at 30 min and 1 day. Even at 7 days after rFVIIa administration, coagulant-active rFVIIa levels in the lung and bone joint isolates from EPCR-over-expressing mice were 2- and 3.75-fold higher, respectively, than those of wild-type counterparts. It may be pertinent to note here that although caution was taken in making tissue homogenates by a controlled standard procedure to measure FVIIa antigen and activity levels, it is possible that tissue samples from different mice may have different percentages of vessels. If a fraction of the FVIIa comes from the vessels in our measurements, then it would cause some inherent variation in our measurements. Nonetheless, this would not significantly alter the data.

## Discussion

We have recently shown that FVIIa binds to EPCR on the endothelial cell surface [13] and this binding could result in EPCR-FVIIa activation of PAR1-mediated cell signaling [16] and internalization of FVIIa bound to EPCR [17]. However, the physiological significance of this interaction *in vivo* is not precisely known. In the present study, by employing EPCR-deficient and EPCR-over-expressing mice, along with wild-type, we provide convincing evidence that rFVIIa administered to mice readily binds to EPCR on the vascular endothelium and thereby reducing the circulating rFVIIa concentration immediately after rFVIIa administration. More importantly, our data show that FVIIa binding to EPCR facilitates the entry of FVIIa to extravascular tissues and the FVIIa redistributed to these compartments remained functionally active and stayed much longer there than in circulation. These data may have significant relevance to hemostasis as well as clinical treatment as rFVIIa is widely used to treat bleeding disorders associated with hemophiliacs with inhibitors as well as other bleeding complications.

The data presented in the manuscript clearly show that exogenously administered rFVIIa associates with EPCR on the endothelium even in the presence of endogenous circulating protein C, the primary ligand for EPCR. The extent of rFVIIa association with endothelium soon after its administration strongly correlates with the degree of EPCR expression on the endothelium. Endothelial and perivascular association of rFVIIa with the vasculature remained more perceptible for somewhat longer periods of time in EPCR-over-expressing mice compared with wild-type mice; nonetheless, an association of FVIIa with the endothelium decreased time-wise in all mice and was negligible 1 day after rFVIIa administration. Negligible staining of FVIIa on the vessels of EPCR-deficient mice suggest that if FVIIa specifically binds the endothelium at additional sites other than EPCR, such binding may be of lower affinity and constitutes a relatively minor fraction. The observations that an EPCR-dependent association of FVIIa with the endothelium is most pronounced soon after its administration and subsequently declines time-wise, along with the retention of FVIIa in tissue homogenates for at least 7 days, suggest that FVIIa bound to the endothelium effectively enters tissues in an EPCR-dependent manner and retained there for longer time periods. It may be pertinent to note here that in unrelated experiments we found no perceptible differences in endothelial permeability at basal conditions among these mice, which rules out the possibility of disparate extravasation as the result of variation in vascular integrity.

An EPCR-dependent transport mechanism of circulating, procoagulant FVIIa from the bloodstream to extravascular tissues may have implications in both physiological and pathophysiological circumstances. In this study, the use of pharmacological concentrations of rFVIIa and EPCR-over expressing mice has provided robustness in detecting the FVIIa transport mechanism. Nonetheless, it is likely that EPCR-dependent transcytosis of FVII/FVIIa operates in normal physiology where low but physiologically meaningful amounts of FVII or FVIIa are transported from the circulation to extravascular compartments. This mechanism would permit the formation of basal TF–FVIIa complexes that are potential primers of the coagulant and signaling responses, which could contribute to hemostasis and vascular integrity. It has been previously shown that TF–FVIIa complexes may form continuously in the adventitia of blood vessels [26,27]. More recently, FVII–TF complexes were demonstrated around dermal vessels in the absence of injury [28]. It is possible that EPCR-mediated FVII transcytosis could be responsible for the above-reported observations of the presence of TF–FVIIa complexes in adventitia. Although EPCR appears to play a critical role in FVIIa transcytosis and accumulation of FVIIa in tissues, a small amount of FVIIa may be distributed to tissues independent of EPCR as we found a small and gradual increase in FVIIa even in EPCR-deficient mice. Further, our earlier studies indicate that a small fraction of FVIIa may associate with the endothelium independent of EPCR [13,19]. Although this binding appears to be specific, at present no receptor other than EPCR has been definitively identified for FVIIa on endothelial cells.

The importance of EPCR-dependent redistribution and the accumulation of extravascular FVIIa assume immediate clinical relevance in terms of therapeutic and prophylactic use in the treatment of bleeding disorders. First, it opens the possibility that the plasma concentration of rFVIIa measured after its administration may not be fully indicative of its bioavailability and subsequent hemostatic efficacy. It is possible that a slow release of rFVIIa bound to EPCR on the endothelium or from tissues back into the circulation, although not readily detectable in blood, could prolong the hemostatic effect of rFVIIa. It is also possible that rFVIIa–TF complexes formed on extravascular cell surfaces after EPCR-mediated transport could contribute to hemostasis in hemophilic patients even in the absence of rFVIIa in the circulation. Second, it could provide rationale for the prophylactic use of rFVIIa. In spite of having a circulatory half-life in the blood of approximately 2–3 h in humans, clinical evidence obtained from hemophilic patients show that once-daily administration of rFVIIa is capable of preventing bleeding episodes during the treatment period [9,12]. Interestingly, the hemostatic effect of rFVIIa not only significantly surpassed the short circulatory half-life of rFVIIa during the treatment period, but can also prevent hemorrhagic events during a 3-month post-treatment period [12]. FVIIa binding to EPCR on the endothelium may result in a long-term benefit by protecting the integrity of the vascular endothelium directly via an EPCR-FVIIa-dependent signaling mechanism [16] or indirectly via facilitating a FVIIa interaction with extravascular TF, which in turn would lead to TF-FVIIa signaling and/or thrombin generation. The present observation that FVIIa, at least pharmacologically administered rFVIIa, binds to EPCR thereby promoting rFVIIa entry into the extravascular tissue may also have important implications on the development of second-generation rFVIIa derivatives for therapeutic purposes. For example, if one considers that the interaction of FVIIa with EPCR plays a role in hemostasis either by direct or indirect mechanisms [29–31], then rFVIIa derivatives that fail to interact with EPCR, such as PEGylated rFVIIa [32], would not be a good therapeutic option even if they were engineered to have prolonged circulatory half-lives [33]. Conversely, if one believes that the hemostatic effect of rFVIIa solely stems from circulating rFVIIa, then engineering a rFVIIa derivate that does not bind to EPCR would prevent sequestration of rFVIIa on the endothelium and thus increase the plasma concentration of rFVIIa by about 30%.

The transcytosis of coagulation factors may have far-reaching implications in hemostasis and vascular biology as the extravascular reservoirs of coagulation factors may contribute to hemostasis and cell signaling. However, at present there is little information on potential mechanisms by which these factors enter the extravasculature space in the absence of injury to blood vessel. The present study puts forward an EPCR-dependent mechanism by which circulating FVIIa is sequestered to the vascular endothelium, redistributed to extravascular tissues and functionally retained for significant periods of time. In terms of pharmacological application of rFVIIa, the present study demonstrates that the plasma concentration may not be accurately indicative of the bioavailability of i.v. injected rFVIIa and functionally active rFVIIa may remain in tissue for a longer period. This possibility in the human system may at least to some degree explain the prolonged hemostatic efficacy of rFVIIa in prophylaxis, as well as place importance on the functional relevance of extravascularly compartmentalized FVIIa. Further studies are needed to fully evaluate the physiological significance of the present finding.

## Addendum

C. Clark performed the majority of experiments described in the manuscript, analyzed the data and prepared the preliminary draft of the manuscript. R. Vatsyayan performed some of the experiments described in the manuscript, helped in exsanguinations of mice and immunohistochemistry. C.T. Esmon provided key reagents and EPCR transgenic mice. U. Hedner and U.R. Pendurthi participated in the study design and contributed to the preparation of the manuscript. L.V.M. Rao designed and reviewed the research, analyzed the data and wrote the manuscript. All authors read the manuscript and contributed to the preparation of the final version of the manuscript.
